# Cyprinid Herpesvirus 2 miR-C12 Attenuates Virus-Mediated Apoptosis and Promotes Virus Propagation by Targeting *Caspase 8*

**DOI:** 10.3389/fmicb.2019.02923

**Published:** 2019-12-18

**Authors:** Jianfei Lu, Zhaoyuan Shen, Liqun Lu, Dan Xu

**Affiliations:** ^1^State Key Laboratory for Managing Biotic and Chemical Threats to the Quality and Safety of Agro-Products, Ningbo University, Ningbo, China; ^2^Laboratory of Biochemistry and Molecular Biology, School of Marine Sciences, Ningbo University, Ningbo, China; ^3^Key Laboratory of Applied Marine Biotechnology of Ministry of Education, Ningbo University, Ningbo, China; ^4^National Pathogen Collection Center for Aquatic Animals, Shanghai Ocean University, Shanghai, China; ^5^Key Laboratory of Aquaculture Ministry for Freshwater Aquatic Genetic Resources, Shanghai Ocean University, Shanghai, China; ^6^National Experimental Teaching Demonstration Center for Fishery Sciences, Shanghai Ocean University, Shanghai, China; ^7^Guangxi Key Laboratory for Marine Biotechnology, Guangxi Institute of Oceanography, Guangxi Academy of Sciences, Beihai, China

**Keywords:** Cyprinid herpesvirus 2, miR-C12, caspase 8, apoptosis, propagation

## Abstract

DNA viruses, most notably members of the herpesvirus family, generally encode miRNAs to mediate both virus and host genes expression. We previously demonstrated that Cyprinid herpesvirus 2 (CyHV-2) encodes 17 miRNAs that are involved in innate immune signaling pathways. In this study, the function of CyHV-2-encoded miRNA was further investigated in GiCF cells. We found that miR-C4 promoted CyHV-2-induced apoptosis, while miR-C12 decreased CyHV-2-induced apoptosis. miR-C12 targeted to 3′ UTR sequence of *caspase 8* and suppressed the expression of *caspase 8*. Besides, the silencing of caspase 8 by specific siRNA led to the attenuation of CyHV-2-induced apoptosis. Furthermore, caspase 8 was downregulated in cells transfected with miR-C12 during CyHV-2 infection. Overexpression of miR-C12 significantly suppressed CyHV-2-induced apoptosis, while silencing of miR-C12 promoted CyHV-2-induced apoptosis. Finally, inhibition of miR-C12 resulted in suppression of CyHV-2 propagation, overexpression of miR-C12, and CASP8-siRNA-1 facilitated CyHV-2 propagation. Taken together, our results demonstrated that CyHV-2-encoded miR-C12 to suppress virus-induced apoptosis and promoted virus replication by targeting *caspase 8*.

## Introduction

The *Alloherpesviridae* is a family of double-stranded DNA viruses that infect amphibians and fish, which comprises of four genera: *Batrachovirus*, *Cyprinivirus*, *Icta-lurivirus*, and *Salmonivirus*. In *Cyprinivirus* genus, Cyprinid herpesvirus 1 (CyHV-1), Cyprinid herpesvirus 2 (CyHV-2), and Cyprinid herpesvirus 3 (CyHV-3) infect common carp, crucian carp, or goldfish (Davison et al., 2013). Many reports have demonstrated that *Cyprinivirus*, mainly CyHV-2 and CyHV-3, can lead to latent infection (Reed et al., 2014; Wei et al., 2019), produce virus-encoded MicroRNAs (Donohoe et al., 2015; Lu et al., 2017), and facilitate viral invasion by exploiting various intracellular signaling pathways (Adamek et al., 2014; Zhou et al., 2019). However, the molecular mechanism employed by CyHV-2 is largely unknown.

Apoptosis, or programmed cell death, is an energy-dependent process of cell suicide (Huang et al., 2014). Some studies reported that many different herpesviruses regulate apoptosis in infected cells (Miest et al., 2014; Scrochi et al., 2017; You et al., 2017). In our previous study, a novel cell line (Carassius auratus gibelio caudal fin cell line, GiCF) permission to CyHV-2 replication was established, and we demonstrated that CyHV-2 infection induces GiCF cell apoptosis (Lu et al., 2018a).

MicroRNAs (miRNAs), are a class of small non-coding RNAs involved in post-transcriptional regulation of target gene expression in various biological processes (Ambros, 2004). A number of herpesviruses, including CyHV-2 and CyHV-3, encode miRNAs in infected cells. The regulatory effects of miRNAs involved in viral infection include apoptosis, oncogenic transformation, and modulation of viral life cycle (Donohoe et al., 2015; Bhela and Rouse, 2017; Lu et al., 2017). Viral miRNAs play an important role in the regulation of cell apoptosis, mostly by decreasing pro-apoptotic or anti-apoptotic genes expression (Pfeffer et al., 2004). For example, BCL2-associated X (BAX) was demonstrated to be a target of viral miRNA ebv-miR-BART16 (Marquitz et al., 2011); BCL2-associated death promoter (BAD) and the pro-apoptotic protein caspase 3 are targets of the ebv-miR-BART20-5p, ebv-miR-BART16 or ebv-miR-BART1-3p, respectively (Vereide et al., 2014). Our previous studies have identified 17 viral miRNAs from CyHV-2-infected crucian carp kidney, and have shown that the host genes PIN1, IRF3, and RBMX are the targets of CyHV-2-encoded miR-C4 (Lu et al., 2017). Recently, it was reported that host miRNAs are involved in CyHV-2 infection and participate in the regulation of apoptosis and immune-related genes (Lu et al., 2018b). However, whether CyHV-2-encoded miRNA has a regulatory effect on CyHV-2-induced apoptosis is still unknown.

Here, we report that miR-C12 encoded by CyHV-2 suppresses virus-induced apoptosis and promotes virus replication by targeting *caspase 8*. We show that overexpression of miR-C12 reduces the expression of *caspase 8* and inhibits CyHV-2-induced apoptosis. In contrast, inhibition of miR-C12 promotes CyHV-2-induced apoptosis. We also demonstrate that downregulation of *caspase 8* results in exacerbated CyHV-2 replication, while upregulation of *caspase 8* results in CyHV-2 replication suppression. These results reveal that miR-C12 regulates CyHV-2-induced apoptosis through *caspase 8*.

## Materials and Methods

### Cells and Virus

*C. auratus gibelio* caudal fin (GiCF) cell line was established in our previous work (Lu et al., 2018a), cells were grown in M199 medium (Gibco, USA) with 10% fetal bovine serum (Gibco, USA) and antibiotics (100 U penicillin ml^−1^ and 100 mg streptomycin ml^−1^) at 25°C. Hela cells were cultured in MEM Medium (Gibco, USA) supplemented with 10% fetal bovine serum (Gibco, USA) at 37°C and 5% CO_2_. The CyHV-2 strain was isolated from infected *C. auratus gibelio* samples cultured in Sheyang City, Jiangsu Province, China (Xu et al., 2014).

### Flow Cytometry Assay

Detection of cell apoptosis was conducted as previously described (Lu et al., 2018a). Briefly, cells were digested by 0.25% trypsin and stained for 20 min in the dark at room temperature with the Muse Annexin V and Dead Cell Reagent (Merck Millipore, USA). The stained cells was analyzed by Muse Cell Analyzer (Merck Millipore, USA), at least 10,000 events were collected for the cell gate.

### miRNA Mimics and Inhibitors

All the miRNA mimics (dsRNA oligonucleotides) and miRNA inhibitors were commercially synthesized by Shanghai GenePharma (Shanghai, China) and the sequences were in Table 1. All the miRNA mimics, miR-NC, miR-C12 inhibitor, and inhibitor NC were transfected using RNAiMAX reagent (Invitrogen, USA).

**Table 1:** Oligonucleotide primers used for amplifying cDNAs, expressing constructs, and gene expression analysis.

Name	Sense	Antisense
**Oligonucleotides for siRNA**		
CASP8-siRNA-1	GCUUAGACUUGCUUGGAAUTT	AUUCCAAGCAAGUCUAAGCTT
CASP8-siRNA-2	GCAGAUUGUUUCGCAGAAUTT	AUUCUGCGAAACAAUCUGCTT
CASP8-siRNA-3	GCCUUACACUAGCAUCCAATT	UUGGAUGCUAGUGUAAGGCTT
**CASP8 3′ UTR primer**		
CASP8 WT	GAATTCCGGCCACCATGAAGCAGATGCCTG	CGGGATCCCGCGCAGTAAACTTTAACTCAA
CASP8 MT	TTGCTGTATCTGTGCGTCTGATCTTAATTA	TAATTAAGATCAGACGCACAGATACAGCAA
**qPCR primers**		
CASP8-q	CTGTTTTGGGCGTGGATG	CCTTGGCAGGCTTGAATG
CyHV-2-q	TTAGCGTCAGGTCCATAG	GGCGTGTAGAAATCAAACT
β-actin	CACTGTGCCCATCTACGAG	CCATCTCCTGCTCGAAGTC
**Oligonucleotides for miRNA mimics**		
miR-C4 mimics	UGUUUUAUCCGCGAGUACUUU	AGUACUCGCGGAUAAAACAUU
miR-C5 mimics	AUGUACCCGCGGAUGAAGCAUC	UGCUUCAUCCGCGGGUACAUUU
miR-C6	ACGUCUCGCCGGGGAGACUCU	AGUCUCCCCGGCGAGACGUUU
miR-C10	ACGGCGACUGGAGUCUGAGCGC	GCUCAGACUCCAGUCGCCGUUU
miR-C12 mimics	AGACGCACUCUCGGACAACCAGG	UGGUUGUCCGAGAGUGCGUCUUU
miR-C15	UGGCGUUCAUAGAGGCACUCUU	GAGUGCCUCUAUGAACGCCAUU
miR-C17	AGAACCGUGGACCUGUCUGGAA	CCAGACAGGUCCACGGUUCUUU
Mimics control	UUCUCCGAACGUGUCACGU	ACGUGACACGUUCGGAGAA
**Oligonucleotides for miRNA inhibitor**		
miR-C12 inhibitor	CCUGGUUGUCCGAGAGUGCGUCU	
Inhibitor control	CAGUACUUUUGUGUAGUACAA	

### RNA Interference

siRNAs were commercially synthesized by Shanghai GenePharma (Shanghai, China), and the siRNAs sequences were listed in Table 1. All the siRNAs were transfected into GiCF cells with 50 nM of each oligonucleotides using RNAiMAX reagent.

### Plasmids

The 3′ UTRs of *caspase 8* were amplified from GiCF cDNA, digested with *Smal* and *Xhol*, and ligated into pGL3-Basic Dual-Luciferase Vector (Promega, USA). Point mutation was conducted by the Fast Site-Directed Mutagenesis Kit (Tiangen, China) and the PCR primers were listed in Table 1. All the enzymes used for plasmid construction were produced by Takara (Dalian, China). The constructed plasmid sequences were verified by Sangon (Shanghai, China). Co-transfections with small RNA and pGL3 plasmids were performed with Lipofectamine 3000 (Invitrogen, USA).

### Luciferase Assays

Hela cells transfected with pGL3 reporter plasmids and miR-C12 mimics, miR-NC, miR-C12 inhibitor, or inhibitor NC were analyzed post-transfection. The luciferase assays were conducted as previously described (Lu et al., 2017). The Dual-Glo luciferase assay kit (Promega, USA) was used to detect the quantity of firefly and Renilla luciferase by GloMax-Multi Detection System (Promega, USA). The relative luciferase activity was normalized to the levels in the renilla luciferase controls.

### Western Blot Analysis

The cell extracts were lysed in lysis buffer (50 mM Tris-HCl, 150 mM NaCl, 0.1% SDS, 1% Triton X-100, 1 mM phenylmethylsulfonyl fluoride, pH 7.8). Proteins were separated by 10% SDS-PAGE, followed by transfer to PVDF membranes (GE Healthcare). Membrane was incubated overnight with primary antibody. After that, the membrane was incubated with secondary antibody conjugated with alkaline phosphatase (1:5,000) (Abmart, China). Finally, the membrane was conducted by ECL Western blot analysis kit (Thermo Fisher, USA). The optical density of the bands was quantified using TanonImage software (Tanon, China), caspase 8, or ORF72 protein expression was normalized to that of β-actin, representative blots of three independent experiments are shown.

Caspase 8 polyclonal antibody was custom-made in GL Biochem Ltd. (Shanghai, China) by Peptides N2 (DHQKLHEIDEDLTST) and C7(RGHKQMPEPRYTLTKK). β-actin monoclonal antibody was supplied by Abmart (Wuhan, China), monoclonal antibody of ORF72 was produced in previous studies (Kong et al., 2017).

### Real-Time Quantitative PCR

For mRNA quantification, total RNA was extracted using the TRIzol reagent (Invitrogen, CA, USA) following the manufacturer’s instructions. Total RNA was reverse transcribed using the PrimeScript RT Master Mix (Takara, Japan). qRT-PCR was performed using the SYBR Premix Ex Taq (Takara), β-actin was used as an internal standard, the primers were listed in Table 1. At least, three independent biological replicates were used for each gene. All reactions were performed in triplicate on the CFX96 Real-time PCR Detection System (Bio-Rad, USA). Expression of the different genes was analyzed by 2^−ΔΔCT^ method.

The miRNA quantification was analyzed by Stem-loop qRT-PCR as described previously (Chen et al., 2005). The Hairpin-it™ MicroRNAs Quantitation PCR Kit custom-made in GenePharma was performed based on vendor protocols. Briefly, total RNA was first extracted using TRIzol reagent (Invitrogen, USA). For qRT-PCR, 1 μg of total RNA was reverse transcribed, and qPCR amplification was conducted in a 20 μl reaction. Synthetic pure miRNA was used as the standard. qPCR data normalized to total RNA was used to determine the miRNA copy number per 1 μg of total RNA. All the samples were carried out in triplicate and the data are represented as the mean ± SD.

### Caspase 3/7 Activity

Caspase 3/7 activity of GiCF cells was conducted by caspase-Glo 3/7 assay kit (Promega, USA). In brief, GiCF cells were collected and washed with PBS, mixed the cells with Caspase-Glo 3/7 reagent, and incubated for 1 h at room temperature in the dark. Subsequently, the mixture was measured using a SPECTRA MAX 190 (Molecular Devices, USA).

### Detection of CyHV-2 Copies

The detection of CyHV-2 copies was performed as described previously (Xu et al., 2014). Total DNA was extracted using a Tissue Genomic DNA Isolation Kit (Tiangen, China), 1 μl of genomic DNA was used for qPCR analysis. The qPCR conditions were as follows: 95°C for 30 s, followed by 40 cycles at 95°C for 5 s, 55°C for 30 s, and 95°C for 10 s, 65°C for 5 s, 95°C for 5 s. Real-time assays were performed in a CFX96™ Real-Time PCR Detection System (Bio-Rad, USA).

### Statistical Analysis

All data were analyzed by one-way ANOVA to calculate the means and standard deviations for triplicate assays, and the data were conducted using Student’s *t*-test and a *p* <0.05 or < 0.01 were considered statistically significant.

## Results

### Effect of Viral miRNA on CyHV-2-Induced Cell Apoptosis

**Figure 1:**
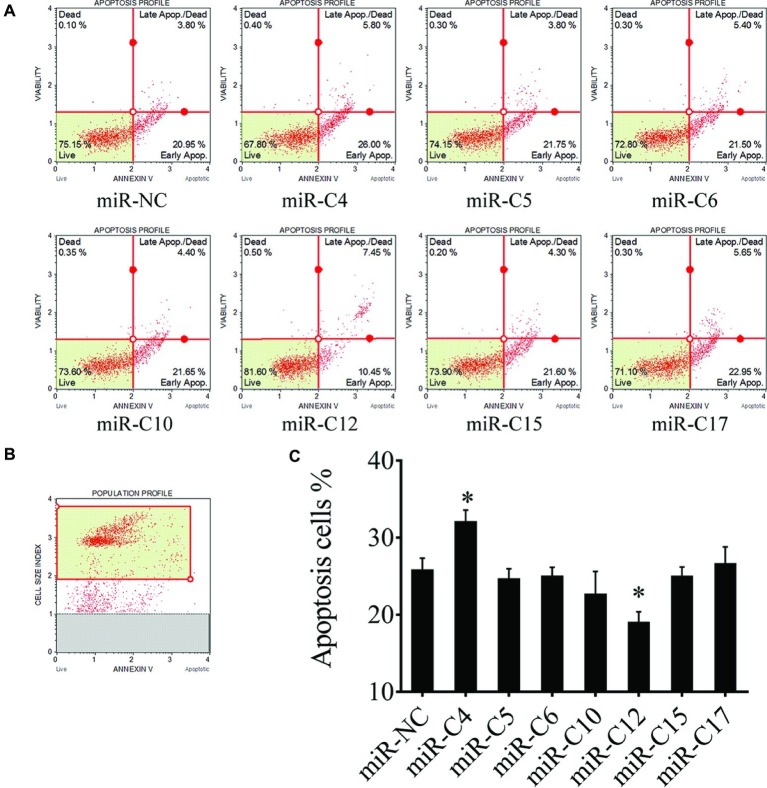
Effect of viral miRNAs on CyHV-2-induced apoptosis. GiCF cells were infected with CyHV-2 (MOI = 0.1), transfected with eight different miRNA mimics 1 h post infection. Cell apoptosis at 24 h post-transfection was determined. **(A)** Apoptotic cells were quantified by flow cytometry at 24 h post-transfection. **(B)** Scatter plots of flow cytometry, cells in the red box were used for subsequent analysis. **(C)** Statistical the percentages of apoptosis. Data represent the means for three independent experiments, error bars are the standard errors. *p < 0.05.

Viral miRNAs play important roles in regulating cell apoptosis and CyHV-2 infection triggers apoptosis in GiCF cells. To investigate the role of CyHV-2 miRNAs in regulating cell apoptosis, seven relatively high-expressed CyHV-2 miRNAs were selected to examine their effects on CyHV-2-induced apoptosis. As shown in Figures 1A–C, miR-C4 promoted CyHV-2-induced cell apoptosis (by 24.9%), while miR-C12 decreased CyHV-2-induced apoptosis (by 27.14%).

### miR-C12 Targets the 3′ UTR of *Caspase 8*

**Figure 2:**
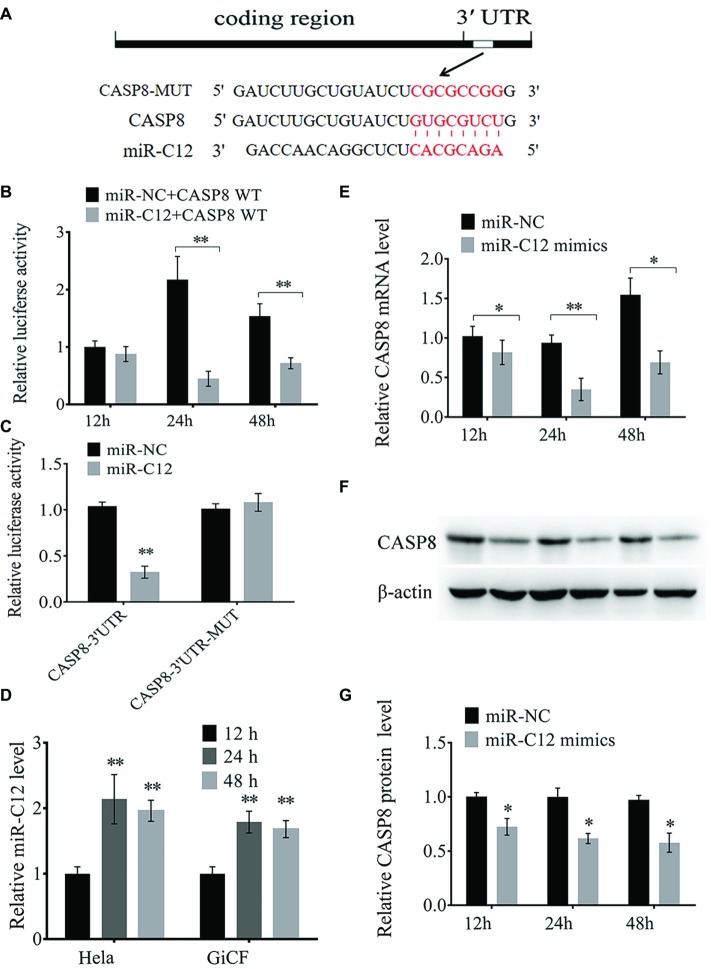
miR-C12 directly targets the 3′ UTR of *caspase 8* mRNA. **(A)** Sequence of putative binding site of miR-C12 within 3′ UTR of CASP8 mRNA. Mutations were introduced to the binding site. **(B)** HeLa cells were co-transfected with miR-C12 mimics or control miRNA and CASP8-WT. Following 12, 24, and 48 h post-transfection, luciferase activity was determined. **(C)** HeLa cells were transfected with miR-C12 mimics or control miRNA, together with CASP8-WT or CASP8-MT. Luciferase activity was determined 24 h post-transfection. **(D)** Relative expression level of miR-C12 post-transfection of miR-C12 mimics in HeLa cells. **(E)** The effect of miR-C12 on endogenous CASP8. GiCF cells were transfected with miR-C12 mimics or miR-NC, the expression level of CASP8 was determined by real-time qRT-PCR and Western blot **(F)** at 12, 24, and 48 h post-transfection. **(G)** Bar graphs displaying the CASP8 protein level. Data were represented as the means ± SD of three independent experiments performed in triplicate. **p < 0.01; *p < 0.05.

Next, we determined the mechanism of miR-C4 and miR-C12 in CyHV-2-induced cell apoptosis. In our previous research, *caspase 8* was predicted as one of the candidate target gene of miR-C12, and we did not find a target gene directly related to apoptosis in miR-C4 (Lu et al., 2017). Therefore, miR-C12 was the main research object of this study. The binding site of miR-C12 to *caspase 8* was shown in Figure 2A. The prediction results suggest that miR-C12 may be involved in the regulation of cell apoptosis.

To verify the predicted results, a dual-luciferase reporter assay was performed in HeLa cells. As shown in Figure 2B, the relative luciferase activity following co-transfection with miR-C12 mimics compared to transfection with control miRNAs (miR-NC) was reduced by 53.26% at 48 h and 79.42% at 24 h post-transfection, with no significant change in the 12 h post-transfection group. However, no significant change in luciferase was observed in cells transfected with mutant-type constructs 24 h post-transfection (Figure 2C). Besides, miR-C12 expression level was increased by 2.14-fold on 24 h and 1.96-fold on 48 h in miR-C12 mimics transfected HeLa cells compared to 12 h post-transfection. In addition, miR-C12 expression level was increased by 1.79-fold on 24 h and 1.68-fold on 48 h in miR-C12 mimics transfected GiCF (Figure 2D).

We next investigated the mechanism responsible for the suppression of *caspase 8* by miR-C12 in GiCF cells. To this end, we transfected miR-C12 mimics or control miRNA into GiCF cells and measured the mRNA and protein levels of caspase 8 in 12, 24, and 48 h post-transfection. As shown in Figures 2E–G, miR-C12 mimics transfection significantly decreased *caspase 8* mRNA by 80.13% in 12 h, 37.25% in 24 h, and 44.76% in 48 h compared to miR-NC group, caspase 8 protein level was reduced by 70.66% in 12, 62.96 h in 24 h, and 57.86% in 48 h compared to miR-NC group (Figure 2D).

These findings suggest that miR-C12 targets 3′ UTR of *caspase 8* and may play a key role in cell apoptosis.

### Caspase 8 Enhances CyHV-2-Induced Cell Apoptosis

**Figure 3:**
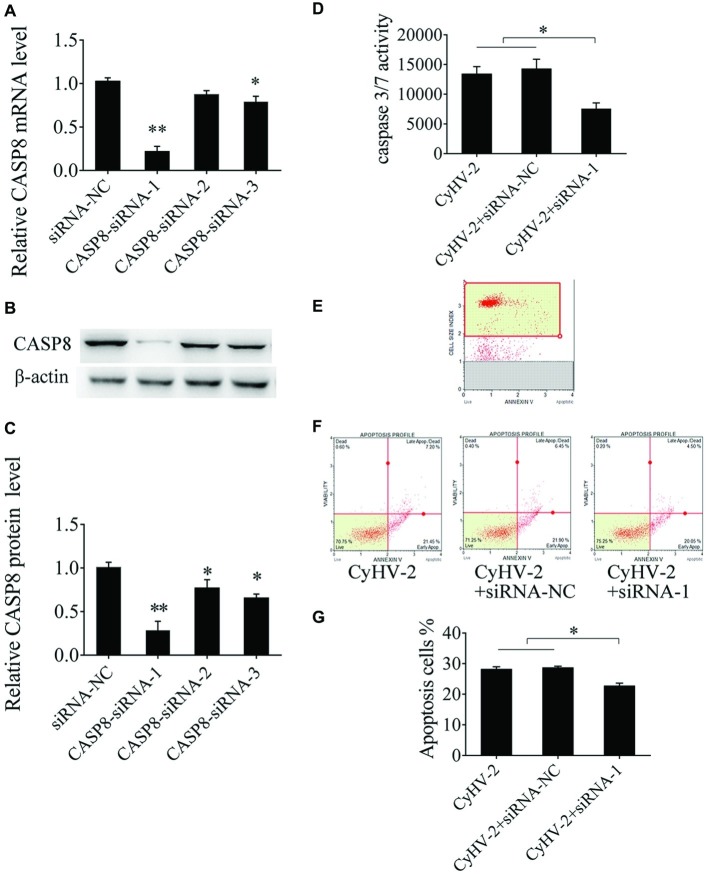
Effect of caspase 8 on CyHV-2-mediated apoptosis. **(A,B)** Silencing of caspase 8 in GiCF cells by specific siRNA. GiCF cells were transfected with different CASP8 siRNA (CASP8-siRNA-1, CASP8-siRNA-2, and CASP8-siRNA-3) or siRNA NC. After 24 h, CASP8 mRNA levels were detected by qRT-PCR **(A)**, and protein level was measured by western blot analysis. **(C)** Bar graphs displaying the CASP8 protein level post-transfection of siRNA. **(D)** *Caspase 8* was downregulated by CASP8-siRNA-1 in virus-infected GiCF cells post infection, and caspase-3/7 activity was assayed using Caspase-Glo 3/7 assay kit. **(E)** Scatter plots of flow cytometry. **(F)** Apoptotic cells were assayed using annexin V and caspase-3/7 activity assay **(G)**. Data represent the means for three independent experiments. **p < 0.01; *p < 0.05.

Caspase 8 is required for apoptosis and plays crucial role in the antiviral immune response in mammalian cells (Mocarski et al., 2011). However, there is little known about the function of caspase 8 in teleost. Here, we aimed to study caspase 8 function in CyHV-2-induced apoptosis. Sequence-specific siRNA was transfected to GiCF cells, qRT-PCR showed that CASP8-siRNA-1 reduced *caspase 8* mRNA by 79.14%, *p* < 0.01 (Figure 3A). Western blot analysis showed that CASP8-siRNA-1 decreased caspase 8 protein by 72.14%, *p* < 0.01 (Figures 3B,C). To further examine the function of caspase 8 on CyHV-2-induced apoptosis, caspase 8 was downregulated by CASP8-siRNA-1 followed by CyHV-2 infection, and the apoptotic activity was then determined. The data indicated that silencing of caspase 8 lead to decline of caspase 3/7 activity (0.43-fold) (Figure 3D) and the ratio of apoptotic cells (0.19-fold) (Figures 3E–G).

### miR-C12 Downregulates Expression of Caspase 8

**Figure 4:**
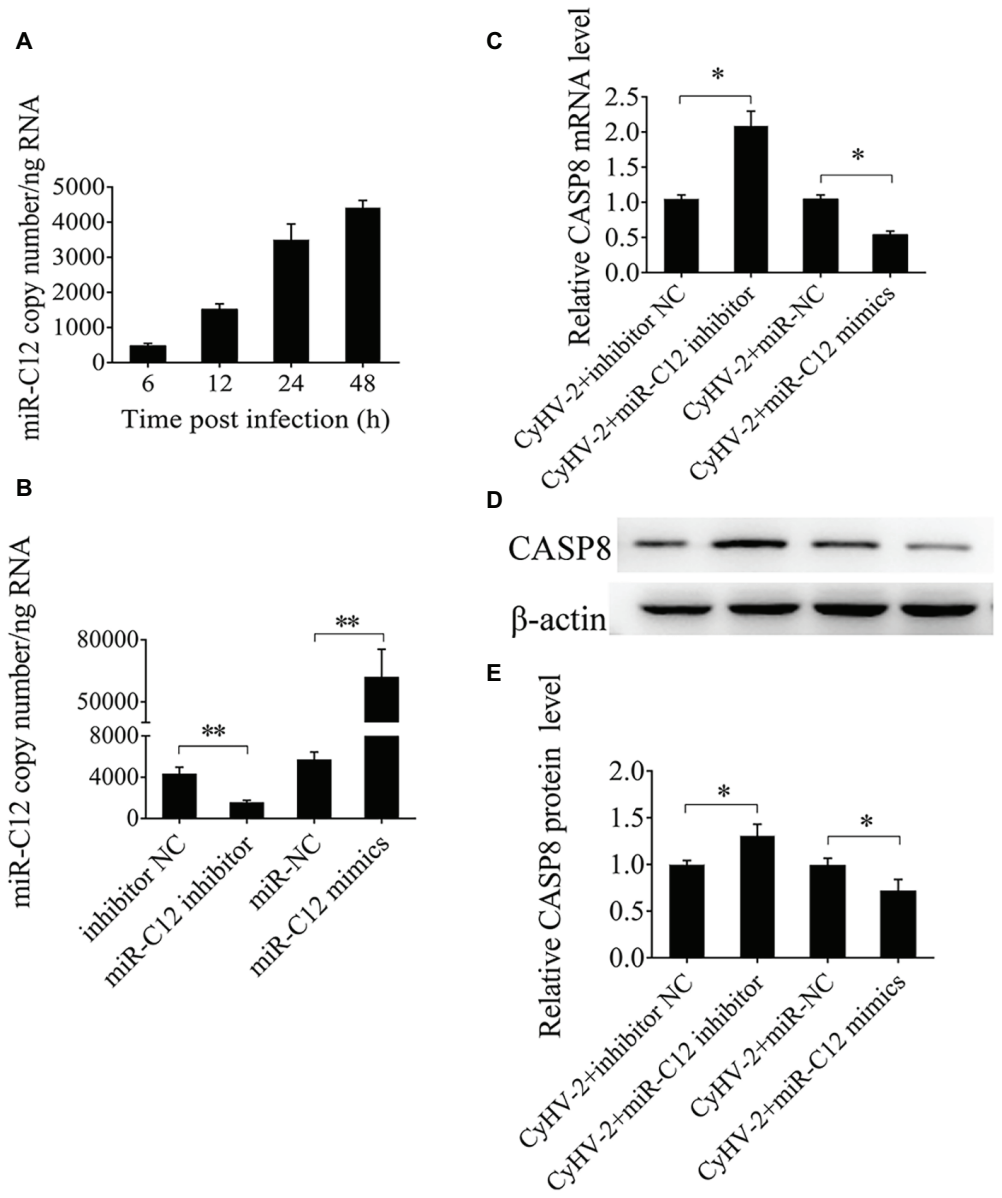
Effect of miR-C12 on caspase 8 expression. **(A)** Expression of miR-C12 in CyHV-2-infected GiCF cells. miR-C12 expression levels at 6, 12, 24, and 48 h post infection was measured by qRT-PCR. **(B)** Effects of the miR-C12 inhibitor or miR-C12 mimics on miR-C12 expression. miR-C12 expression levels were quantified by qRT-PCR at 24 h post-transfection in CyHV-2-infected GiCF (MOI = 0.1) cells transfected with the miR-C12 inhibitor and the inhibitor negative control or miR-C12 mimics and miR-NC. **(C)** Effect of miR-C12 on *caspase 8* mRNA expression in CyHV-2-infected GiCF cells. qRT-PCR detection of caspase 8 in CyHV-2-infected GiCF cells treated with the miR-C12 mimic or miR-C12 inhibitor. **(D,E)** Effect of miR-C12 on caspase 8 protein expression. Western blot detection of caspase 8 in CyHV-2-infected GiCF cells. The miRNA negative control and inhibitor negative control were included in the experiments, β-actin was used as a control. **p < 0.01; *p < 0.05.

To evaluate the effect of miR-C12 on caspase 8 expression, expression of miR-C12 was quantified during CyHV-2 infection by qRT-PCR. The results reveal that miR-C12 was detected from 6 h post CyHV-2 infection, and miR-C12 expression increased with the time of CyHV-2 infection (Figure 4A). Inhibiting miR-C12 with the miR-C12 inhibitor results in downregulation of miR-C12 expression in CyHV-2-infected GiCF cells (0.65-fold), over expression of miR-C12 mimics led to the increase of miR-C12 expression by 19.75-fold compared to the miRNA control group in CyHV-2-infected GiCF cells (Figure 4B). Next, miR-C12 mimics and inhibitor were used to evaluate the impact of overexpression or inhibition of miR-C12 on the expression of caspase 8 during CyHV-2 infection. As expected, the results shown that miR-C12 overexpression led to inhibition of *caspase 8* mRNA levels by 0.56-fold and 0.39-fold in caspase 8 protein, miR-C12 inhibitor increased *caspase 8* mRNA (2.19-fold) and protein levels (1.36-fold) at 24 h post infection (Figures 4C–E). The above data suggest that miR-C12 downregulates expression of caspase 8 during CyHV-2 infection.

### miR-C12 Attenuates CyHV-2-Induced Apoptosis

Given that miR-C12 decreases CyHV-2-induced cell apoptosis and inhibits the expression of *caspase 8*, inhibition of caspase 8 decreases CyHV-2-mediated apoptosis, we hypothesized that miR-C12 attenuates virus-mediated apoptosis by targeting *caspase 8*.

**Figure 5:**
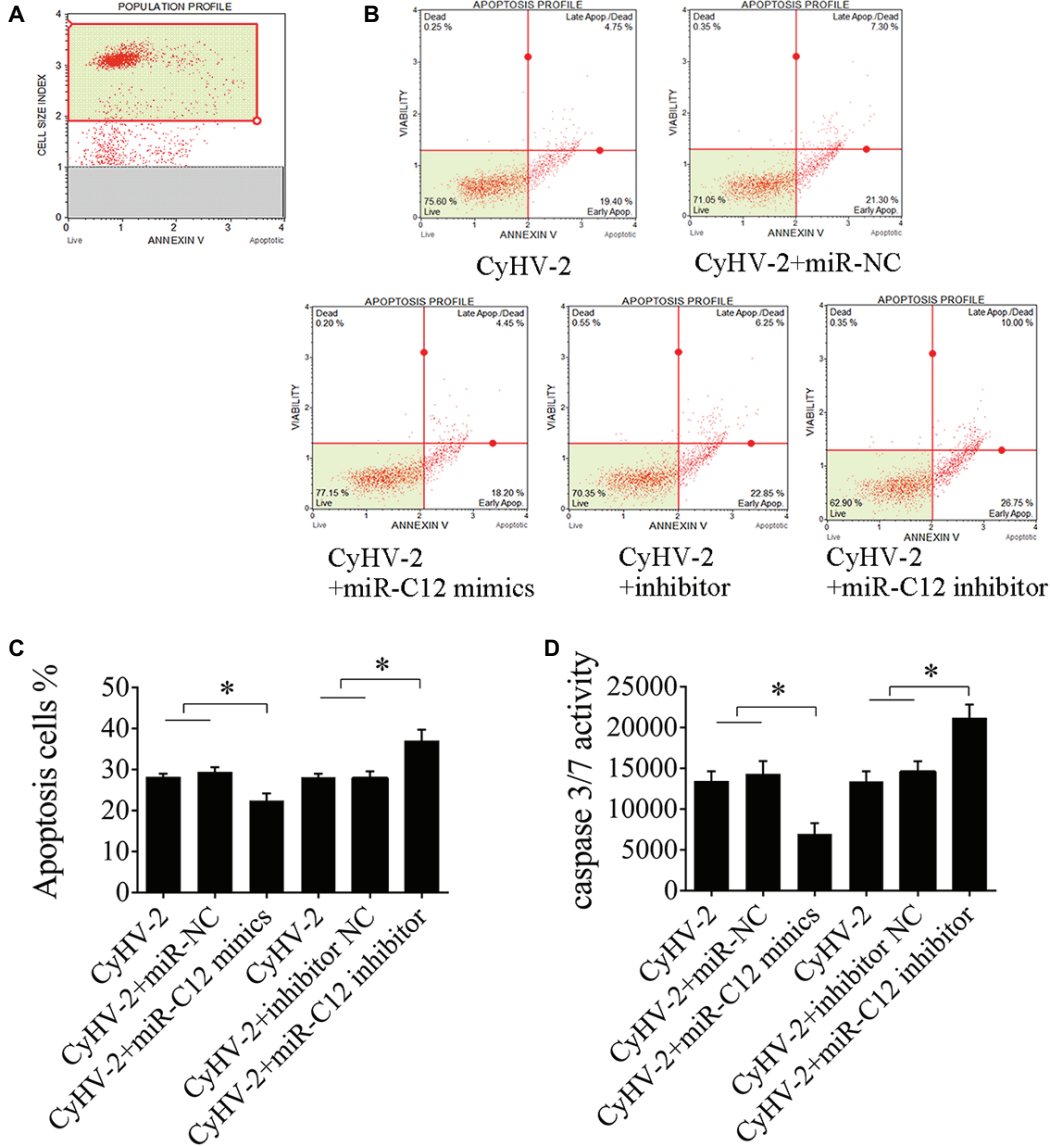
Effect of miR-C12 on CyHV-2-mediated apoptosis. **(A)** Scatter plots of flow cytometry. miR-C12 was overexpressed or knocked down with the miR-C12 inhibitor in virus-infected GiCF cells. At 24 h post infection, apoptosis was assayed using annexin V analysis **(B,C)** and caspase 3/7 activity analysis **(D)**. Data represents the means ± SD from three independent experiments. **p < 0.01; *p < 0.05.

To prove this hypothesis, we investigated the effect of miR-C12 on CyHV-2-induced cell apoptosis. Here, apoptosis was evaluated under conditions of miR-C12 knock down or overexpression. Annexin V analysis demonstrated that miR-C12 overexpression resulted in an inhibition of CyHV-2-induced cell apoptosis at 24 h post infection (0.25-fold). However, silencing miR-C12 with miR-C12 inhibitor resulted in an increase in CyHV-2-induced cell apoptosis (1.28-fold) (Figures 5A–C). In addition, caspase 3/7 activity was decreased in miR-C12 mimic transfected cell (0.51-fold), and promoted in miR-C12 inhibitor transfected cell (1.45-fold) (Figure 5D). The data demonstrated that miR-C12 plays a negative role in CyHV-2-induced apoptosis.

### Role of miR-C12 in the CyHV-2 Propagation

**Figure 6:**
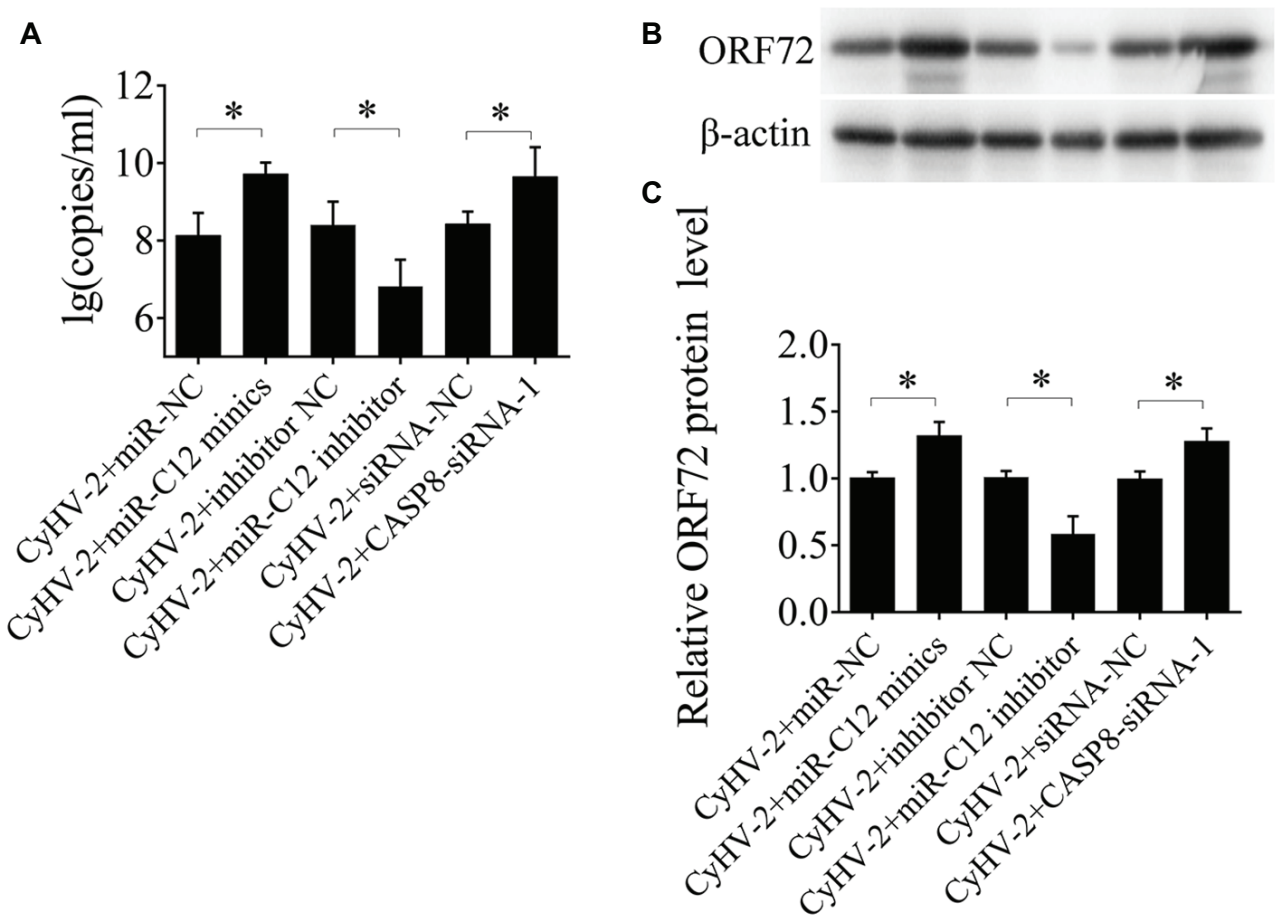
Role of miR-C12 in the CyHV-2 propagation. GiCF cells were infected with CyHV-2 (MOI = 0.1) and transfected with miR-NC, miR-C12 mimics, inhibitor NC, miR-C12 inhibitor, siRNA-NC, or CASP8-siRNA-1 2 h post infection. The infected cells were then collected and subjected to qRT-PCR **(A)** or Western blot **(B)** to detect CyHV-2 propagation. **(C)** Bar graphs displaying the ORF72 protein level.

To assess the effects of miR-C12 on CyHV-2 propagation, miR-C12 was silenced with miR-C12 inhibitor or overexpressed with miR-C12 mimics. At 24 h post infection, CyHV-2 copies in the miR-C12 inhibitor treated cells were decreased by 97.3% compared to the control group (1.2-fold). miR-C12 mimics led to the increase of CyHV-2 copies by 41.69-fold compared to the miRNA control group. miR-C12 mimics led to the increase of CyHV-2 copies by 41.69-fold compared to the miRNA control group. CASP8-siRNA-1 led to the increase of CyHV-2 copies by 16.6-fold compared to the control group (Figure 6A). Besides, as a major capsid protein of CyHV-2, the expression trend of ORF72 protein was consistent with CyHV-2 copies. miR-C12 mimics and CASP8-siRNA-1 promoted ORF72 protein level by 1.42- and 1.39-fold, miR-C12 inhibitor treat resulted in an decrease of ORF72 protein level (0.54-fold) (Figures 6B,C). These findings indicate that CyHV-2 encodes miR-C12 to facilitate its propagation.

### Model of miR-C12 Function in CyHV-2 Infection

**Figure 7:**
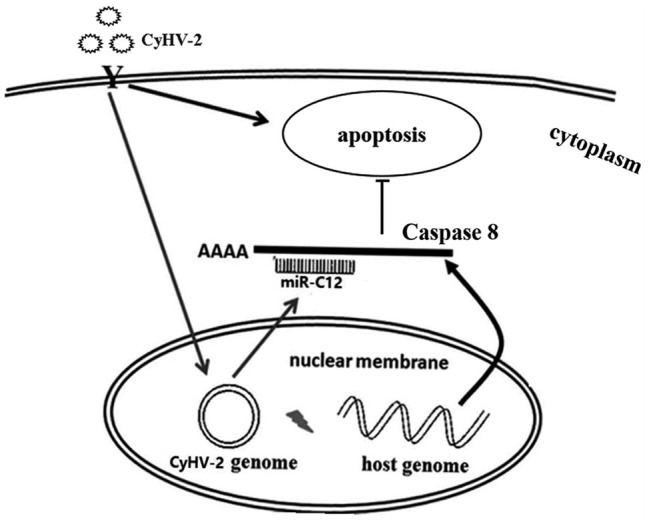
Summary of the functions of miR-C12 in CyHV-2 infection. CyHV-2 encodes miR-C12 post infection, miR-C12 inhibits CyHV-2-induced apoptosis by targeting *caspase 8*. Subsequently, the inhibition of CyHV-2-induced apoptosis led to the increase of CyHV-2 propagation.

Taken together, proposed model of miR-C12 in CyHV-2 infection is shown in Figure 7. Our studies demonstrated that CyHV-2 encodes miR-C12 and attenuates CyHV-2-induced apoptosis by targeting *caspase 8*. Subsequently, the inhibition of CyHV-2-induced apoptosis led to the increase of CyHV-2 propagation.

## Discussion

The aim of this study was to investigate the role of CyHV-2-encoded miRNA in CyHV-2-induced apoptosis. In this study, we found that miR-C4 promotes CyHV-2-induced apoptosis, while miR-C12 decreases CyHV-2-induced apoptosis. We also found that miR-C12 is employed by CyHV-2 to facilitate viral replication. Above all, our results demonstrate that CyHV-2-encoded miR-C12 suppress virus-induced apoptosis and promotes virus replication by targeting *caspase 8*.

A number of herpesviruses, especially gamaherpesvirus, have evolved strategies to encode viral miRNAs during infection, counter host immunity through miRNA (Guo et al., 2015). Many virus-encoded miRNAs target the host gene mRNAs or viral genes to mediate the gene regulation function (Kincaid and Sullivan, 2012). Here, we found that miR-C4 promotes CyHV-2-induced cell apoptosis, while miR-C12 decreases CyHV-2-induced apoptosis. To promote infection, virus-encoded miRNAs mediate virus infection by targeting to host functional genes. There are several reasons for the opposite regulation of miR-C4 and miR-C12 in CyHV-2-induced cell apoptosis. Firstly, miRNA was found to have a profound effect on the expression pattern of several hundred mRNAs (Reddy, 2015). One miRNA may target and regulate multiple genes, and one gene can be regulated by multiple miRNA (Qian et al., 2017). For example, several miRNAs in EBV cluster 1 have been identified to show both pro-apoptotic and anti-apoptotic effects (Vereide et al., 2014). Secondly, circular RNAs (circRNAs) or long noncoding RNA (LncRNA) interacted with miRNA, can reduce miRNA regulatory effect on mRNAs (Reddy, 2015; Kulcheski et al., 2016). miR-C4 might regulate CyHV-2-induced apoptosis through these unknown circRNAs or LncRNAs. Further work is necessary to clarify the exact mechanism of CyHV-2 miRNAs in virus-induced apoptosis. Besides, due to the limit of sequence depth and the absence of crucian carp genomic information, genes used to predict miRNA targets were incomplete. miR-C4 might regulate CyHV-2-induced apoptosis through unknown genes.

Viruses have evolved various strategies to repress apoptosis by regulating pro-apoptotic and anti-apoptotic factors. During its life cycle, α-herpesvirus utilizes various anti-apoptotic strategies to suppress programmed cell death (You et al., 2017). Viral miRNAs target both host and viral mRNAs, and regulate virus-induced apoptosis by targeting to pro-apoptotic or anti-apoptotic factors. By targeting host mRNAs, viral miRNAs can regulate genes involved in apoptosis, immune response, cell differentiation, cell cycle control, and intracellular trafficking (Piedade and Azevedopereira, 2016). In this study, *caspase 8* was confirmed as a target of miR-C12, and miR-C12 suppresses CyHV-2-induced apoptosis by targeting *caspase 8* in GiCF cells. As an initiator and apical activator caspase, caspase 8 plays a key role in extrinsic apoptosis (Tummers and Green, 2017). Several miRNAs mediate apoptosis by targeting *caspase 8*. For instance, host miR-134 negatively regulates the oligodendrocytes (OLs) apoptosis by targeting *caspase 8* (Xiao et al., 2019). WSSV-encoded WSSV-miR-N24 represses the apoptosis of shrimp hemocytes by targeting *caspase 8* in shrimp hemocytes (Huang et al., 2014). In conclusion, to facilitate viral infection, CyHV-2-encoded miR-C12 to suppress virus-induced apoptosis by targeting to *caspase 8*.

Several reports have shown that virus-encoded miRNAs influence virus replication and propagation. Viral miRNAs are expressed during infection, suggesting that viral miRNA might participate in virus propagation and survival (Qian et al., 2017). For example, WSSV-miR-N24 inhibits cell apoptosis through increase in *caspase 8* expression, leading to an increase in WSSV copy number (Huang et al., 2014). Furthermore, kshv-miR-K12-5 and kshv-miR-K12-9 target BCLAF1, and promote KSHV lytic replication (Bellare and Ganem, 2009). In contrast, host miRNA can inhibit the replication of hepatitis B virus by regulating the expression of a host gene that is beneficial to virus infection (Zhao et al., 2014). Notably, WSSV and KSHV encode miRNAs, suggesting that these viruses have evolved to utilize the expression of viral miRNA to promote their propagation. In this study, we found overexpression of miR-C12 mimics led to a significant increase of CyHV-2 copies, while suppressing miR-C12 with the miR-C12 inhibitor resulted in a decrease in CyHV-2 replication. Caspase 8 increased with CyHV-2 infection and viral miR-C12 decreased apoptosis; this indicated that the CyHV-2 infection increases the expression of caspase 8, and miR-C12 was serving to counteract this effect. The complexity of the miRNA-mRNA interaction was also observed in WSSV- and HIV-encoded miRNA (Klase et al., 2009; Huang et al., 2014). Collectively, our results demonstrate that miR-C12 is employed by CyHV-2 to regulate host *caspase 8* expression to promote viral propagation through inhibition of cell apoptosis.

In summary, our results reveal that CyHV-2 miR-C12 is an important suppressor of CyHV-2-induced apoptosis by downregulating *caspase 8* expression. Therefore, it seems that using miR-C12 can be an effective method for CyHV-2 to control distinct host genes and establish an ideal environment for viral propagation, or prevent CyHV-2-infected cell from increasing host immune responses.

## Data Availability Statement

All datasets generated for this study are included in the article/supplementary material.

## Ethics Statement

All animal work in this paper was conducted according to relevant national and international guidelines. All animal care and experimental procedures were approved by the Committee on Animal Care and Use and the Committee on the Ethics of Animal Experiments of Ningbo University.

## Author Contributions

JL, DX, and LL were responsible for the experimental design, analyses, and interpretation of the data. JL drafted the manuscript. JL and ZS performed the experiments. All authors read and approved the final version of the manuscript.

### Conflict of Interest

The authors declare that the research was conducted in the absence of any commercial or financial relationships that could be construed as a potential conflict of interest.
